# Supplementary material for: Neural processes mediating contextual influences on human choice behaviour
Source: Nat Commun. 2016 Aug 18;7:12416. doi: 10.1038/ncomms12416 (PMC4992127; doi:10.1038/ncomms12416)
Supplement: Supplementary Information — Supplementary Figure 1 and Supplementary Table 1 [file ncomms12416-s1.pdf]

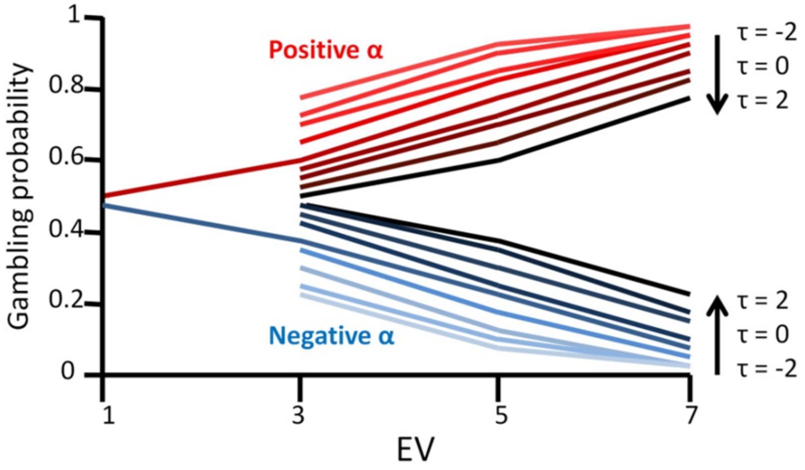

Supplementary Figure 1. Plot of the gambling probability as a function of EV for a set of simulated agents with specific parameters. Red lines represent agents with a positive value function parameter α (equal to 0.1, in red) and blue lines represent agents with a negative Value function parameter α (equal to -0.1, in blue). Behaviour of agents with context parameter τ equal to zero is represented by lines extending from £1 to £7 EV. For the high-value context comprising £3, £5 and £7 EV, agents with different τ are plotted in which τ increases in £0.5 steps from -£2 to £2 along a bright-to-dark gradient.

1) **Activation for first trials of blocks compared to baseline**

Area	Peak coordinates	Z	P
Right hippocampus*	32, -37, -12	4.02	0.003
Left posterior occipital cortex	-18, -80, -7	5.50	0.002

2) **Deactivation for first trials of blocks compared to baseline**

Area	Peak coordinates	Z	P
Left posterior lateral parietal cortex	-58, -22, 25	5.15	0.002
Right ventrolateral prefrontal cortex	57, 10, 10	5.07	0.003
Right inferior frontal gyrus	44, 20, -7	4.93	0.004

3) **Activation for £7 minus £1 EV**

Area	Peak coordinates	Z	P
Left striatum*	-10, 8, -2	5.11	<0.001
Right striatum*	12, 13, 0	5.21	<0.001
VTA/SN*	-8, -17, -15	3.80	0.005
Right posterior occipital cortex	19, -85, -7	5.11	0.009
Right anterior insula	34, 18, -5	4.89	0.036

4) **Activation for £3 and £5 EV for low minus high-value context**

Area	Peak coordinates	Z	P
Left striatum*	-3 10, -10	3.07	0.046
Right striatum*	4, 10, -7	2.87	0.073 n.s.
VTA/SN*	-8, -17, -15	2.40	0.095 n.s.

5) **Activation for difference in £5 minus £3 EV when comparing low minus high-value context**

Area	Peak coordinates	Z	P
VTA/SN*	-3, -24, -22	3.23	0.036

Supplementary Table 1. Regions showing a significant activation for the contrasts. Montreal Neurological Institute (MNI) coordinates are used. For ROIs (marked with asterisks), p statistics are small volume corrected (SVC), while for other areas a correction was performed in relation to the recorded partial volume of the brain. In both, $p < 0.05$ family wise error (FWE) was used as significance threshold. Note that in some cases, statistics for ROIs were not significant (n.s.) but shown a significance trend only; these are reported only for descriptive purposes. We found no regions showing a significant activation for (i) £1 minus £7 EV, (ii) £3 and £5 for the high minus low value context, (iii) the difference for £5 minus £3 EV when comparing high minus low-value context.
